# Green synthesis, characterisation of Au and Ag nanoparticles by various bioextracts and their usability at graphite electrode modification

**DOI:** 10.55730/1300-0527.3432

**Published:** 2022-04-19

**Authors:** Emine YILMAZ ARSLAN, Ender BİÇER, Elif SEKMEN, Semiha ÇAKIR

**Affiliations:** 1 Republic of Turkey Ministry of Health, Turkish Medicines and Medical Devices Agency, Ankara, Turkey; 2 Department of Chemistry, Faculty of Arts and Sciences, Ondokuz Mayıs University, Samsun, Turkey; 3 Republic of Turkey Ministry of Agriculture and Forestry, Ankara, Turkey; 4 Department of Chemistry, Faculty of Sciences, Gazi University, Ankara, Turkey

**Keywords:** Green synthesis, metal nanoparticles, bioextracts, modified graphite electrodes

## Abstract

In this study, the biosynthesis of Au and Ag nanoparticles (AuNp and AgNp) with a green chemistry approach was performed by using bioextracts of various fruits and vegetables (*Lycopersicon esculentum* (LE), *Cucumis sativus* (CS), *Malus domestica* (MD), *Cucurbita pepo* (CP), *Apiumgraveolens var. rapaceum* (AVR), *Prunus cerasifera* (PC), *Oleraceae var. botrytis* (OVB)). In the determination of optimum experimental conditions, the parameters such as the type and concentration of bioextract, concentration of metal ion solutions, the ratio of metal ion solution to bioextract, pH, reaction time and temperature were found to be significant. The characterisation of AuNp and AgNp synthesized by providing the most appropriate experimental conditions was performed by UV-Vis., SEM, EDX, XPS and FTIR techniques. The characterisation study showed that, AuNp and AgNp were successfully synthesized and detailed information was obtained about their stabilities, sizes, homogeneities etc. In the final step, the usability of the green synthesized nanoparticles in the modification of pencil graphite electrodes (PGE) was investigated. Electrochemical characterisation of AuNp/GE and AgNp/GE electrodes was performed using cyclic voltammetry (CV) and electrochemical impedance spectroscopy (EIS) techniques. The obtained results and calculated electrochemical parameters showed that these modified electrodes have better conductivity, electron transfer rate and electrocatalytic activity.

## 1. Introduction

Nanotechnology is occupied with the synthesis and application of materials at nanoscale and is being also used in chemical, biological and medicinal fields [1, 2]. Nanotechnology offers new opportunities for material science. Electrochemistry could be incorporated for the better understanding of its properties. Materials at nanoscale have most interesting properties. Because of size, distribution and morphology, nanoparticles (Nps) exhibit absolutely new or improved properties [3]. MNps have been investigated extensively owing to their catalytic, optic, electronic, magnetic and antibacterial properties [4–9]. Nps have been widely used in various biomedical applications, like in the development of biosensors for health diagnosis, imaging, drug delivery, and therapy, because of the some advantages which easier fabrication procedures, biocompatibility, greater chemical stability, high, conductivity vast electrochemical potential range, high catalytic activity [10–15]. The synthesis of metal nanoparticles (MNps) is an effective research subject because of their biomedical applications [16]. A number of chemical and physical methods used at the synthesis of Nps exhibit various disadvantages such as use of toxic solvents, high-energy consumption, hazardous products etc. Therefore, the development of environment and eco-friendly methods at the synthesis of MNps increase their biological applications [16]. Green processes include the safe and eco-friendly methods for the synthesis of Nps that are considered as alternative ways for other conventional methods [17]. Green synthesis has advantages like as cost-effective and environmental friendships and for it there is no to use harmful chemicals, high pressure and temperature [18, 19].

In this study, the biosynthesis of Au and Ag nanoparticles (AuNp and AgNp) with a green chemistry approach was performed by using bioextracts of various fruits and vegetables (LE, CS, MD, CP, AVR, PC, and OVB) [20]. In our knowledge, although the studies carried out on AuNp and AgNp synthesized using both the physical or chemical methods are widely, green synthesis method is relatively few [21–23].

In the present paper, we reported use of seven different bioextracts to synthesize AuNp and AgNp. The synthesized these MNps were characterized using UV-Vis, SEM, EDX, XPS and FT-IR techniques. We also reported simple green synthesize method for modifying GEs with AuNp and AgNp. Electrochemical characterisation of AuNp/GE and AgNp/GE was performed using CV and EIS techniques. The electroactivities of bare GE and modified GEs with AuNp and AgNp for the electron transfer rate of [Fe(CN)_6_]^3−/4−^ redox couple were investigated. This work aimed to shed new light on the potential application of green synthesized Nps in the electrochemical area as modification of GEs.

## 2. Materials and methods

### 2.1. Reagents and chemicals

HCl, NaOH, KNO_3_, CH_3_COOH, KCl, H_3_PO_4_ and H_2_SO_4_ were purchased from Merck. HAuCl_4_·3H_2_O and AgNO_3_ were obtained from Sigma-Aldrich. Stock solutions of reagents were prepared by deionized and distilled water.

### 2.2. Apparatus

SEM and EDX analyses were performed with the Quanta 400F FE-SEM device in the METU central laboratory, and XPS analyses were performed with the SPECS EA 300 device for the morphological characterisation of AuNp and AgNp. T 80 double beam spectrophotometer device was used in the wavelength range of 200–800 nm for the UV-Vis analyses of the synthesized AuNp and AgNp. Fourier transform infrared (FTIR) spectroscopy analysis was performed with a Mattson 1000 spectrometer. Liquid sample containers were used for solutions and bioextracts containing AuNp and AgNp. EIS measurements were performed with a three-electrode, controlled by PAR Versa STAT 3 potentiostat and VersaStudio software.

### 2.3. Preparation of bioextracts

The bioextracts obtained from various fruits and vegetables (LE, CS, MD, CP, AVR, PC, and OVB) were used for the green synthesis of AuNp and AgNp. After the samples, to be bioextracted, passed from the sorting, cleaning, washing and drying stages, respectively, their pieces which were prepared at certain weights and parts were pureed by appropriate methods. Then, these mixtures were filtered through Whatman No. 1 filter paper and centrifuged at 12,000 rpm for 10 min. The extracts were diluted at the rate determined for each metal and used by adjusting pH. The extracts were stored in amber bottles at low temperatures in the refrigerator for 2 days until used. Some samples were prepared fresh just before use [24].

### 2.4. Green synthesis and characterisation of AuNp and AgNp

Stock solutions of 0.01 or 0.1 M were prepared from HAuCl_4_·3H_2_O and AgNO_3_. With using these stock solutions and bioextracts prepared as described in above, bioextract / metal ion solutions were prepared at the ratios determined separately for each metal (in ratios of 3/7, 5/5 and 7/3 by volume). Metal ion solutions and bioextracts were combined by rapid mixing and mixing was continued. It has been observed that the preparation methods, storage conditions, dilution rates and ambient pH of the stock bioextracts are effective in determining the optimum experimental conditions. For this purpose, bioextracts diluted at the most suitable concentration and pH values were determined for each metal. The synthesis of AuNp and AgNp was carried out at room temperature. Thus, the seven green synthesis methods using different bioextracts for AuNp and AgNp were developed under optimum experimental conditions. The desired pH was adjusted by addition of the appropriate amount of stock 0.1 M NaOH solution. Physical evidence of green synthesis of AuNp and AgNp under the determined optimum experimental conditions was immediately followed visually from the changes in the colours of the solutions containing metal ions. Solutions containing synthesized AuNp and AgNp were stored in the refrigerator at +4 °C for 1–12 months, and their stability was investigated.

### 2.5. Characterisation of AuNp and AgNp

UV-Vis spectroscopy, scanning electron microscope (SEM), energy dispersive X-ray (EDX) spectroscopy, X-ray photoelectron spectrometry (XPS), Fourier transform infrared (FT-IR) spectroscopy were used for the characterisation of AuNp and AgNp synthesized by the green synthesis method.

### 2.6. Preparation of pencil graphite electrode (PGE)

**Figure 1:**
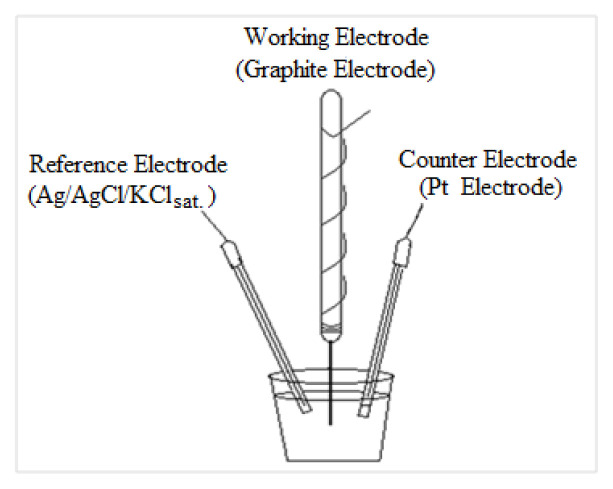
Schematic view of a three-electrode electrochemical cell.

Tombow 0.5 mm 2B pencil tips were used as PGE. It was prepared in such a way that 3.5 cm of the pen tip was left outside and placed as a working electrode in the three-electrode electrochemical cell. It is adjusted so that 1 cm of the pen tip goes into the solution. A thin copper wire is wound around the tip of the pen to ensure conductivity. The electrochemical cell with GE prepared in this way and used as a disposable working electrode is schematised in Figure 1. Activation of GE was carried out in 10 mL of H_3_PO_4_ solution (pH 7) by applying a potential of +1.4 V for 60 s.

### 2.7. Preparation of modified GEs (AuNp/GE and AgNp/GE)

The parts of surface-activated pencil graphite electrodes (PGEs) that are measured and marked are immersed in AuNp and AgNp solutions and kept waiting, allowing the adsorption of AuNp and AgNp to GE surface, so AuNp/GE and AgNp/GE were prepared. These modified electrodes were washed three times with distilled deionized water and stored in a desiccator.

### 2.8. Characterisation of AuNp/GE and AgNp/GE

The characterisation of modification of GEs with AuNp and AgNp synthesized with seven different bioextracts was carried out using EIS and CV electrochemical techniques. At the characterisation study, 0.1 M KCl solution containing 1 × 10^−3^ M [Fe(CN)_6_]^4−^ and [Fe(CN)_6_]^3−^ redox probe was used. Before the EIS experiment, CVs of bare GE and modified GEs with AuNp and AgNp were separately taken. Then, EIS spectra of the modified electrodes taken in the frequency range of 0.05 Hz – 300 kHz and the electrochemical parameters obtained from these spectra were evaluated. Therefore, the results of the characterisation of the modified GE electrodes with AuNp and AgNp were interpreted.

## 3. Results and discussion

### 3.1. Determination of optimum synthesis conditions for AuNp and AgNp

**Figure 2:**
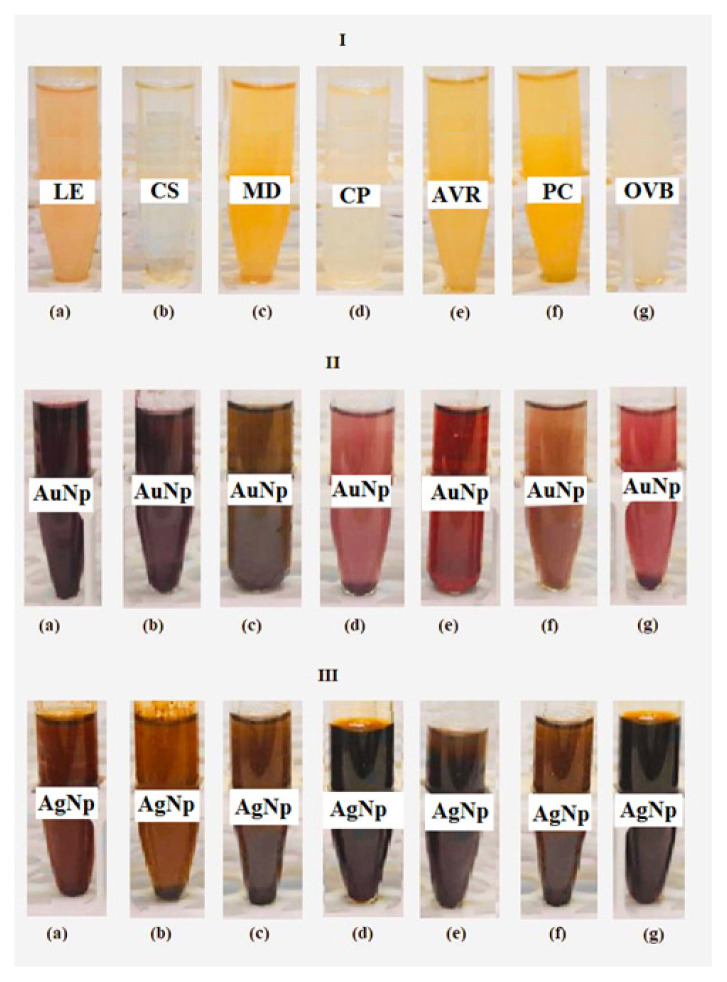
Colours of bioextracts solutions (I) and changes belonging to solutions containing AuNp (II) and AgNp (III) synthesized by bioextracts of a) LE, b) CS, c) MD, d) CP, e) AVR, f) PC, and g) OVB.

At the stable and homogeneous synthesis of AuNp and AgNp, the effects of parameters such as type of bioextract, concentration ratio of bioextract to metal ion solution, pH, reaction time and temperature were investigated. The observation of the colour changes in the solutions showing the formation of AuNp and AgNp facilitated the selection of bioextracts. The colour changes in solutions containing AuNp and AgNp synthesized separately with seven different bioextracts were seen at Figure 2.

As seen in Figure 2, the colours of the solutions containing AuNp and AgNp changed depending on the type of bioextract. The reason for this is that the Np size and shape are different, and it is related to the concentration, type and pH of the bioextract [25].

**Figure 3:**
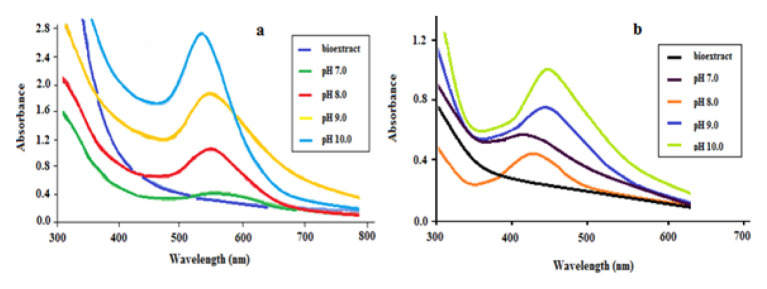
The effect of pH at the green synthesis of a) AuNp, and b) AgNp by LE bioextract.

pH value of the medium is one of the important parameters for the synthesis of AuNp and AgNp. pH affects the yield, stability, size, morphology of the synthesized Nps and also the metal reduction rate of bioextracts [26, 27]. pH of the solutions where the synthesis of AuNp and AgNp was carried out was adjusted with 0.1 M NaOH to values in the range of 7–11 depending on the metal and the type of bioextract. It is striking that the optimum pH values determined for the synthesis are generally high (Figure 3). The reason for this is that the particles have a negative zeta potential, the particles are loaded with a larger negative charge as pH value increases, agglomeration is prevented, and the stability increases [28]. It was observed that pH adjustment affected the synthesis time as well as stability. The colour changes in solutions showing Np formation before pH adjustment are observed in 180 min for AuNp and this period can be up to 24 h or longer for AgNp. When appropriate pH adjustment was made in the same solutions, colour changes indicating the formation of Nps took place immediately.

On the other hand, it was observed that the characteristic surface plasmon resonance (SPR) absorption bands of these MNps were obtained in the first 2–3 min in the UV-Vis spectra of pH-adjusted solutions.

As seen in Figure 3, while the characteristic SPR bands of AuNps and AgNps could not be observed clearly at pH 7, they increased with increasing pH and the maximum increase was obtained at pH 10.

**Figure 4:**
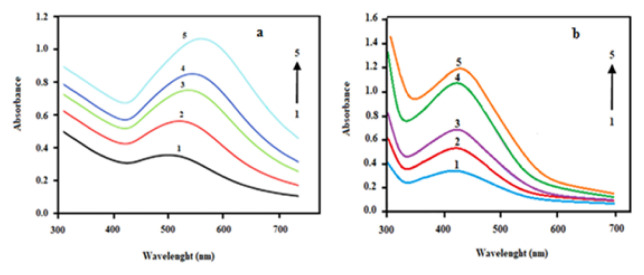
The effect of reaction time on the formation of a) AuNp at concentration ratio of bioextract (LE) to metal of 3:7, b) AgNp at concentration ratio of bioextract (LE) to metal of 5:5 (1) 15, 2) 30, 3) 60, 4) 120, 5) 180 min).

It was observed that the ratio of bioextract to metal and reaction time were at least as important as pH and bioextract type. As given in Figure 4a, when the concentration ratio of bioextract to metal is 3:7, the solution turned light cherry colour in the first 15 min for AuNp and the characteristic SPR absorption band observed in the 500–600 nm range (depending on the particle size) could clearly be obtained. For the synthesis of AgNp, when the concentration ratio of bioextract to metal is 5:5, the solution turned brown colour in the first 15 min and the characteristic SPR absorption band observed in the 400–450 nm range (depending on the particle size) could clearly be obtained (Figure 4b).

Optimum conditions for the synthesis of AuNp and AgNp metal nanoparticles were determined separately. It was observed that the ratio of bioextract to metal and reaction time were at least as important as pH and bioexract type. Therefore, the synthesis of more stable and similar sizes of nanoparticles was carried out by establishing the optimum experimental conditions (as exhibited in Table 1).

### 3.2. Spectroscopic characterisation and surface morphologies of AuNp and AgNp

The formation of AuNp and AgNp was visually noticed by the sudden changes in the colours of metal ion solutions. Also, SEM, EDX, XPS, UV-Vis. and FTIR studies have been carried out to obtain more detailed information about the characterisation of AuNp and AgNp.

#### 3.2.1. UV-Vis spectra of AuNp

**Figure 5:**
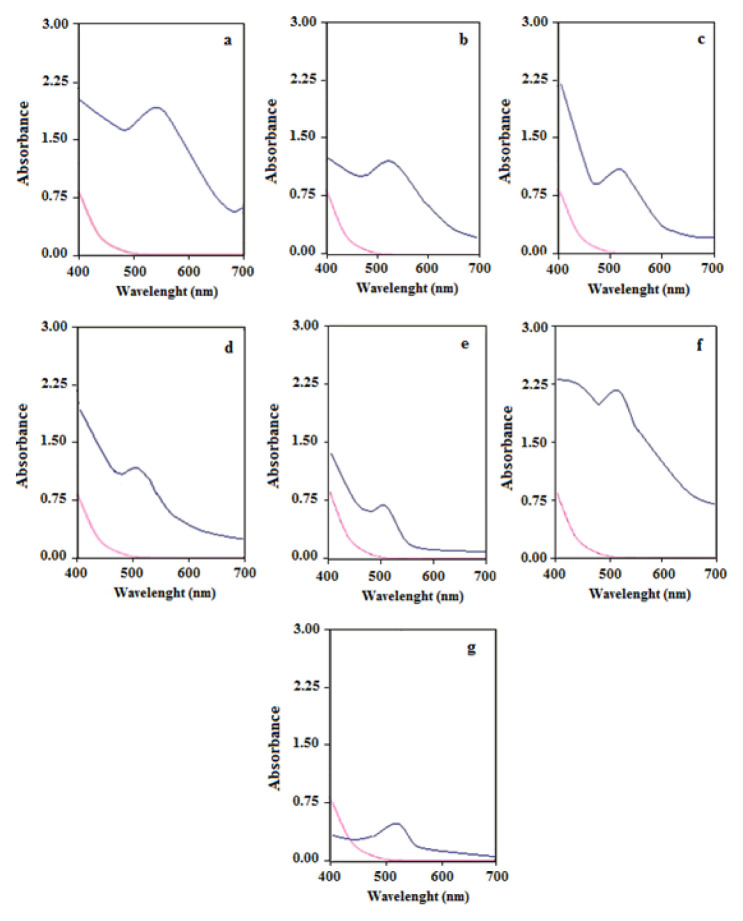
UV-Vis spectra of AuNp solutions (blue line) synthesized with a) LE, b) CS, c) MD, d) CP, e) AVR, f) PC, and g) OVB bioextracts and of Au^3+^ solution (red line).

Seven different green synthesis methods were carried out for AuNp using bioextracts of LE, CS, MD, CP, AVR, PC and OVB. Detailed information on the characterisation of AuNp was obtained by taking the UV-Vis spectra of the solutions containing AuNp synthesized. Observation of the characteristic SPR band of AuNp in UV-Vis spectra not only showed the formation of Np, but also gave detailed information about particle size, shape, stability and homogeneity. Electrons in the conduction band of gold at AuNp form oscillate in the electromagnetic field with the resonance effect of the rays in the visible region. This phenomenon is called SPR. Occurrence of this event is associated with the size of AuNp being smaller than the wavelength of light (d_p_ < λ_light_). Thus, the oscillating free electrons in the conduction band are excited in harmony with the electromagnetic rays in the visible region and characteristic SPR bands are observed in the spectrum. SPR bands provide information about the size and shape of Nps [29]. In the UV-Vis spectra of the solutions containing AuNp in the wavelength range of 200–800 nm, the characteristic SPR band was obtained around 525 ± 15 nm. This SPR band obtained at UV-Vis spectra was found to be in agreement with many other studies in the literature [30,31]. UV-Vis spectra of AuNp synthesized with seven different bioextracts are given in Figure 5.

**Figure 6:**
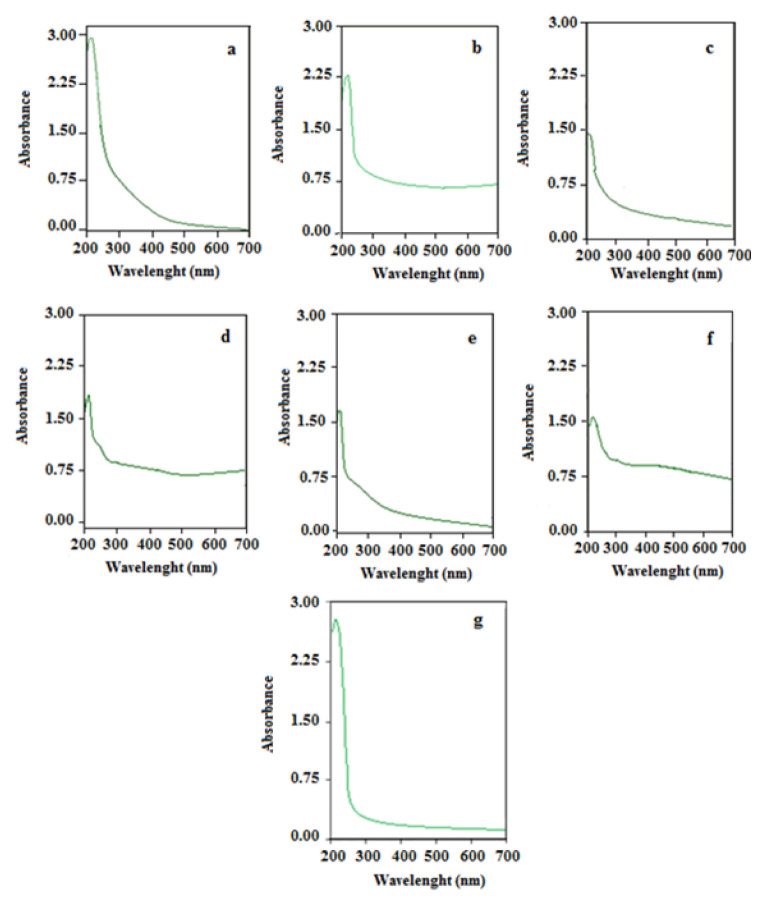
UV-Vis spectra of a) LE, b) CS, c) MD, d) CP, e) AVR, f) PC, and g) OVB bioextracts.

As seen in Figures 5a–5g, small differences at the peak width, wavelength (525 ± 15 nm) and peak height observed on the characteristic SPR bands of AuNp synthesized with seven different bioextracts are due to different particle sizes, shapes and concentrations of Nps [29]. The hue of colours of colloidal solutions of AuNp varies depending on the bioextract used for synthesis and thus the experimental conditions determined for the bioextract. In general, the solution that identifies AuNps is violet in colour. Darkening of the hue (from light violet to dark violet) indicates an increase in Np size. Therefore, the peaks of SPR bands are shifted to longer wavelengths (Figure 5). This finding was supported by SEM images. Changes in the peak height of SPR bands, that is, in absorbance, are related to the number of Nps [32]. In addition, the amount and type of bioextract, and bioextract/metal ratios affect the shape, size and number of the synthesized Nps. Thus, it will play a critical role on the SPR band in the UV-Vis spectra [33]. The UV-Vis spectra of the bioextract solutions used in the synthesis were given in Figure 6.

#### 3.2.2. SEM Study of AuNp

**Figure 7:**
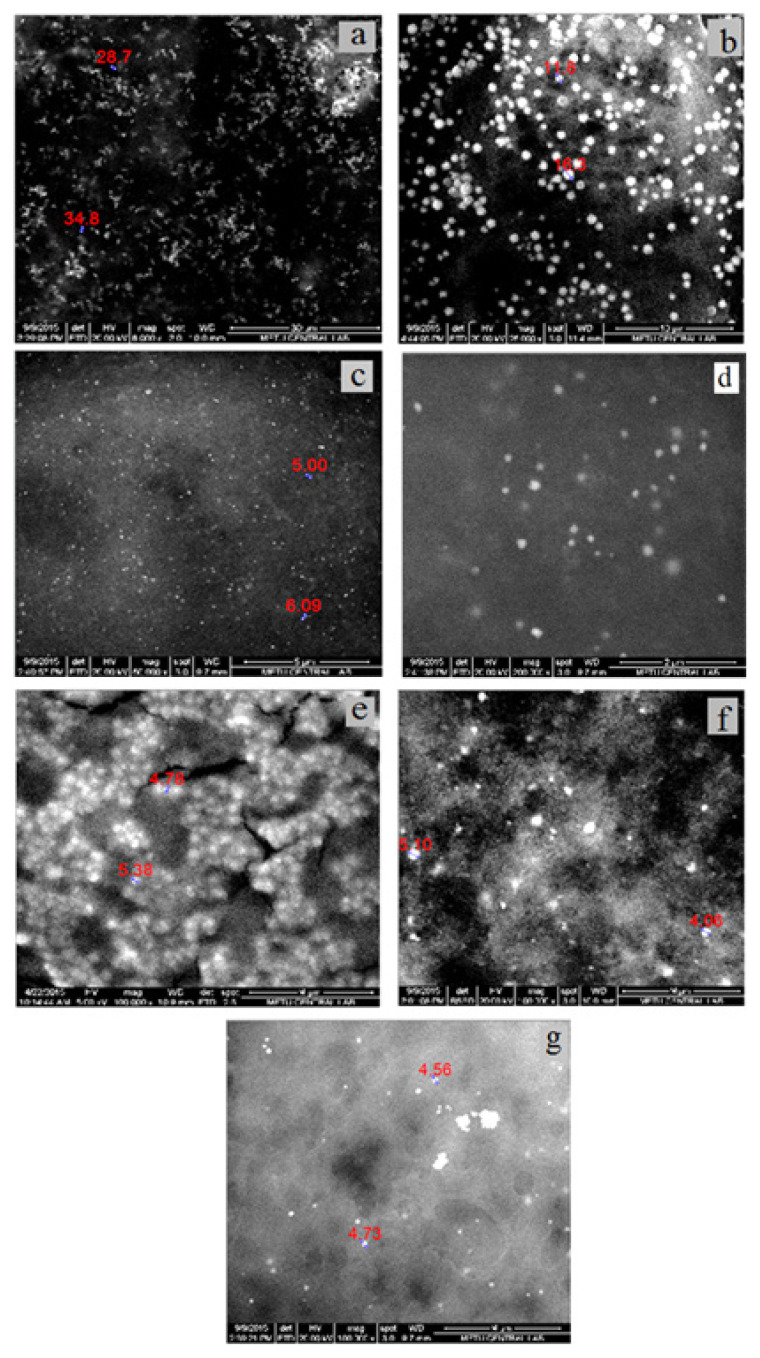
SEM images of AuNp synthesized with a) LE, b) CS, c) MD, d) CP, e) AVR, f) PC, and g) OVB bioextracts.

SEM images of AuNp synthesized with seven different bioextracts are given in Figure 7. From SEM images, the sizes of AuNp were found to be in the range of 5–35 nm. The smallest size Nps were synthesized by CP while the largest sized and nonspherical Nps (25–35 nm) were synthesized by LE. Since the sizes of AuNp synthesized with CP are 5 nm and below this value, a clear measurement could not be taken. As seen in Figure 5, the SPR bands in UV-Vis spectra of AuNps synthesized with LE and CS, which have larger dimensions compared to AuNps synthesized with other extracts, were observed at larger wavelengths (540 and 530 nm, respectively). This result coincides with the red shift of the SPR band as the particle size increases. It is also an expected result that SPR bands of smallest size particles synthesized by CP are at the shortest wavelength (510 nm) (Figure 5). On the other hand, it is another expected result that AuNp solution synthesized with CP in Figure 2 has the lightest colour.

#### 3.2.3. EDX study of AuNp

**Figure 8:**
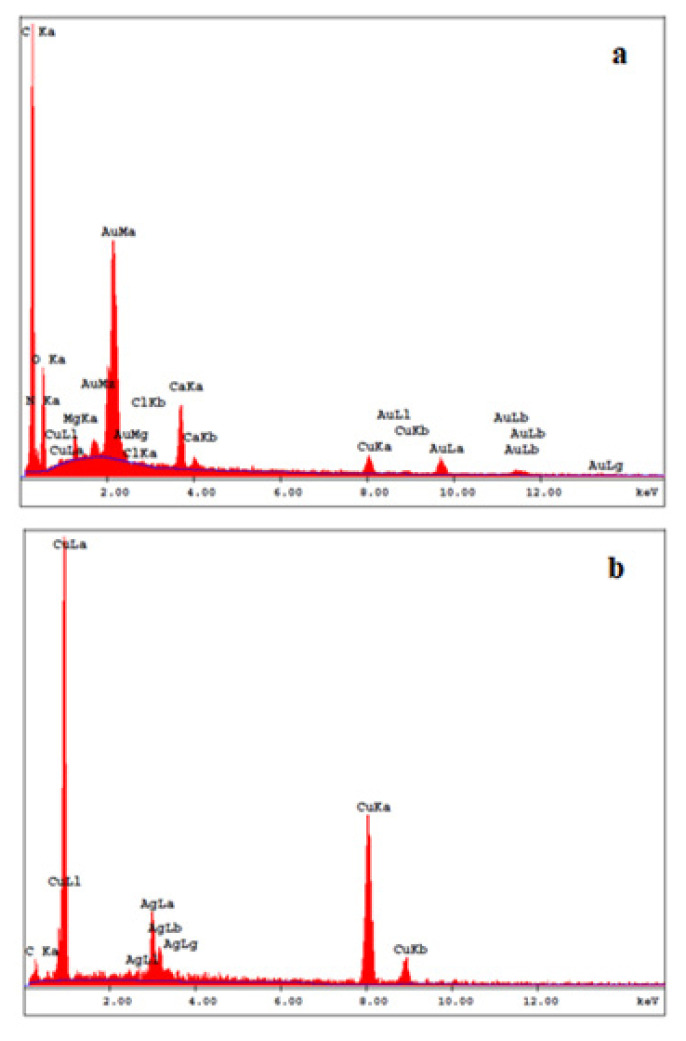
EDX spectrum of a) AuNp synthesized with LE, b) AgNp synthesized with CP.

EDX analysis of AuNp synthesized with LE is given in Figure 8a. On the EDX spectra obtained, the peak showing the formation of AuNp was observed around 2 keV. This finding is in agreement with the literature [34]. The peak at 2 keV was obtained as a result of one of the electrons in the inner orbit of AuNp is detached by the sent electron beam and the characteristic X-rays emitted during the passage of one of the electrons in the other layers to the vacant place. Weak characteristic peaks of C, N, Mg, Na, Ca and O were also observed along with the peaks, showing the formation of AuNps. The peak of Cu is due to copper used to provide conductivity while preparing the sample. The EDX spectra of AuNp synthesized with other bioextracts were also taken and the results were observed to be similar to the characteristic findings in Figure 8a.

#### 3.2.4. XPS study of AuNp

**Figure 9:**
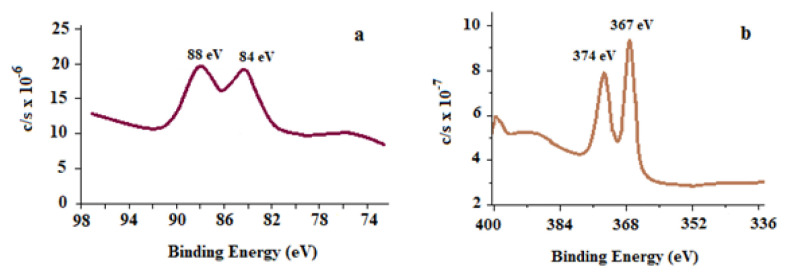
XPS spectrum of a) AuNp synthesized with CS, b) AgNp synthesized with LE.

In the XPS analysis of AuNps, two characteristic gold signals were obtained at 84 and 88 eV, respectively. The signals at 84 and 88 eV are sourced from the binding energies of Au^0^’s electrons with spins 4f_7/2_ and 4f_5/2_, respectively. As an example, XPS spectrum of AuNp synthesized with CS was given in Figure 9a. The fact that these peaks were also observed in XPS spectra of AuNp synthesized with other bioextracts clearly showed that AuNp was successfully synthesized [35, 36].

#### 3.2.5. FTIR spectrum of AuNp

**Figure 10:**
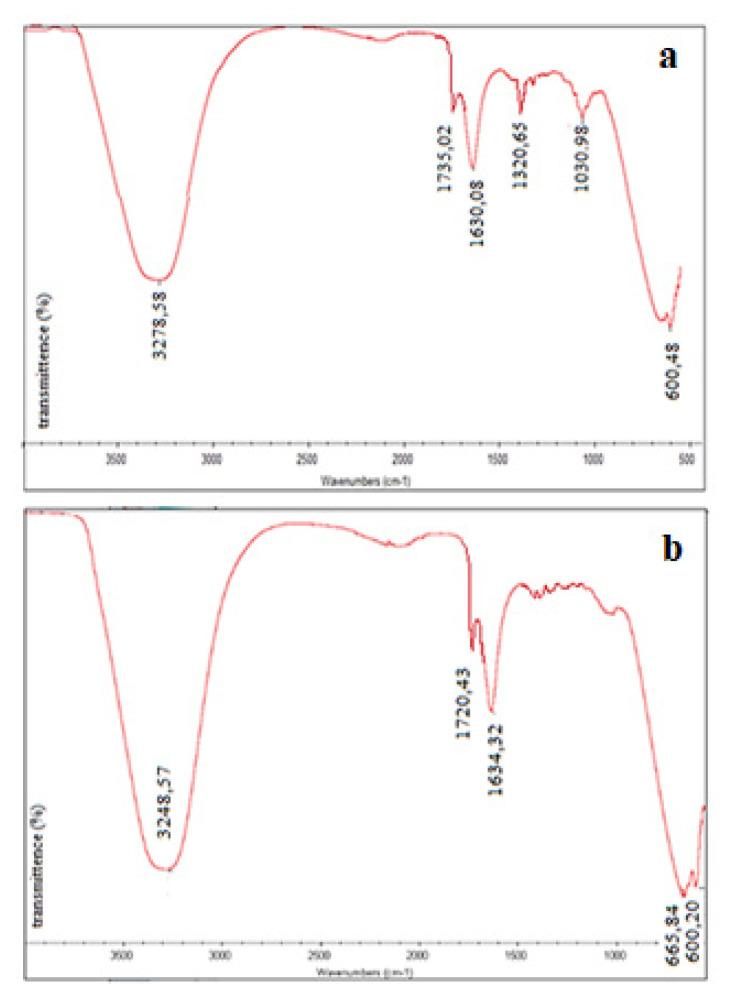
FTIR spectra of MD bioextract (a) and AuNp solution (b).

The characterisation of AuNp were also detailed with the results of FTIR analysis. It was possible to determine the active groups of the reducing molecule of the bioextract and from which group the biologically reduced MNps bind to this biomolecule. For this purpose, as an example, FTIR spectrum of MD bioextract was compared to FTIR spectrum of the solution containing AuNp in Figure 10.

The strong band at 3278.58 cm^−1^ observed in the FTIR spectrum of MD bioextract (Figure 10a) can be attributed to the hydroxyl group. Bands at 1735.02 and 1320.65 cm^−1^ may be assigned to the vibrational movements of the >C=O and C–O groups in the plant metabolite. It is thought that the last groups belong to the carboxylic acid group of acids in the bioextracts. The strong band at 1630.08 cm^−1^ belongs to the vibrational motion of the N–H bond. The band at 1030.98 cm^−1^ was thought to originate from the C–O–C and C–OH functional groups. These vibrational bands are associated with protein bonds in the bioextracts. The band at 600.48 cm^−1^ is due to C–H bending vibrations [37–39]. The frequency of vibrational movement belonging to the >C=O group at the FTIR spectra of solutions containing AuNp synthesized by MD (Figure 10b) shifted from 1735.02 to 1720.43 cm^−1^ (lower frequency). However, the band (1630.08 cm^−1^) belonging to the amide I group (N-H) shifted to a higher frequency (1634.32 cm^−1^). These changes suggest that AuNps are bound from –COOH and N–H groups. In addition, with the synthesis of AuNp, a band of vibrational motion of the >C–O group at 1320.65 cm^−1^ was not observed (Figure 10b) [40].

#### 3.2.6. UV-Vis spectra of AgNp

**Figure 11:**
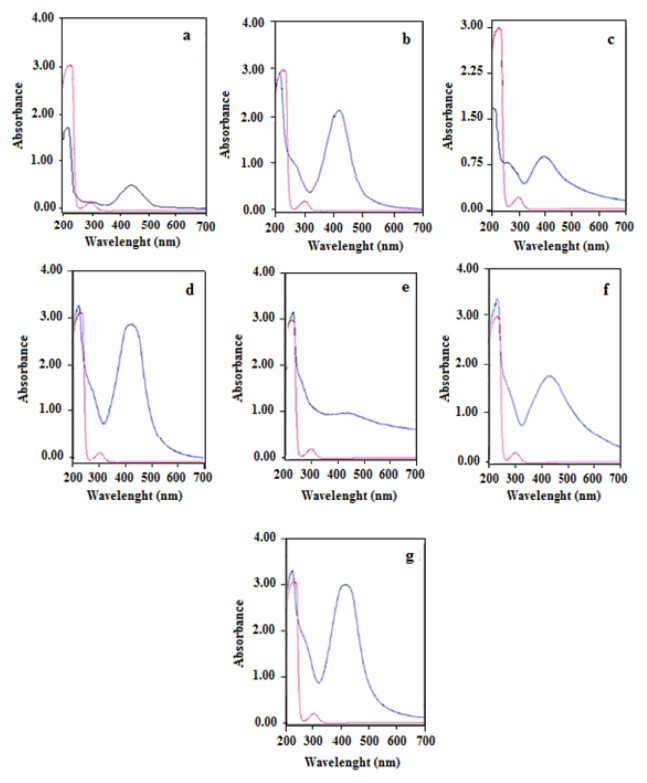
UV-Vis spectra of AgNp solutions (blue line) synthesized with a) LE, b) CS, c) MD, d) CP, e) AVR, f) PC, and g) OVB bioextracts and of Ag^+^ solution (red line).

UV-Vis spectra of AgNp are given in Figure 11. SPR bands are a clear indication of the formation of MNps. In the UV-Vis spectra of AgNp, characteristic SPR bands of AgNp were observed at 425 ± 20 nm. The results of SPR peaks obtained by UV-Vis spectroscopy were consistent with the literature findings. As seen in Figures 11a–11g, small shifts in the wavelengths of SPR bands at which have a maximum absorption, depending on the type of bioextract, are related to the size and shape of Np [32, 41–43]. SPR bands of AgNps synthesized with LE, CS, MD, CP, AVR, PC and OVB bioextracts were observed at wavelengths of 423, 410, 406, 417, 442, 440, and 412 nm, respectively. Due to the large size (30–35 nm) of AgNp synthesized by AVR bioextract, it is expected that its SPR band shifts to a longer wavelength (442 nm) with compared to the others.

#### 3.2.7. SEM analysis of AgNp

**Figure 12:**
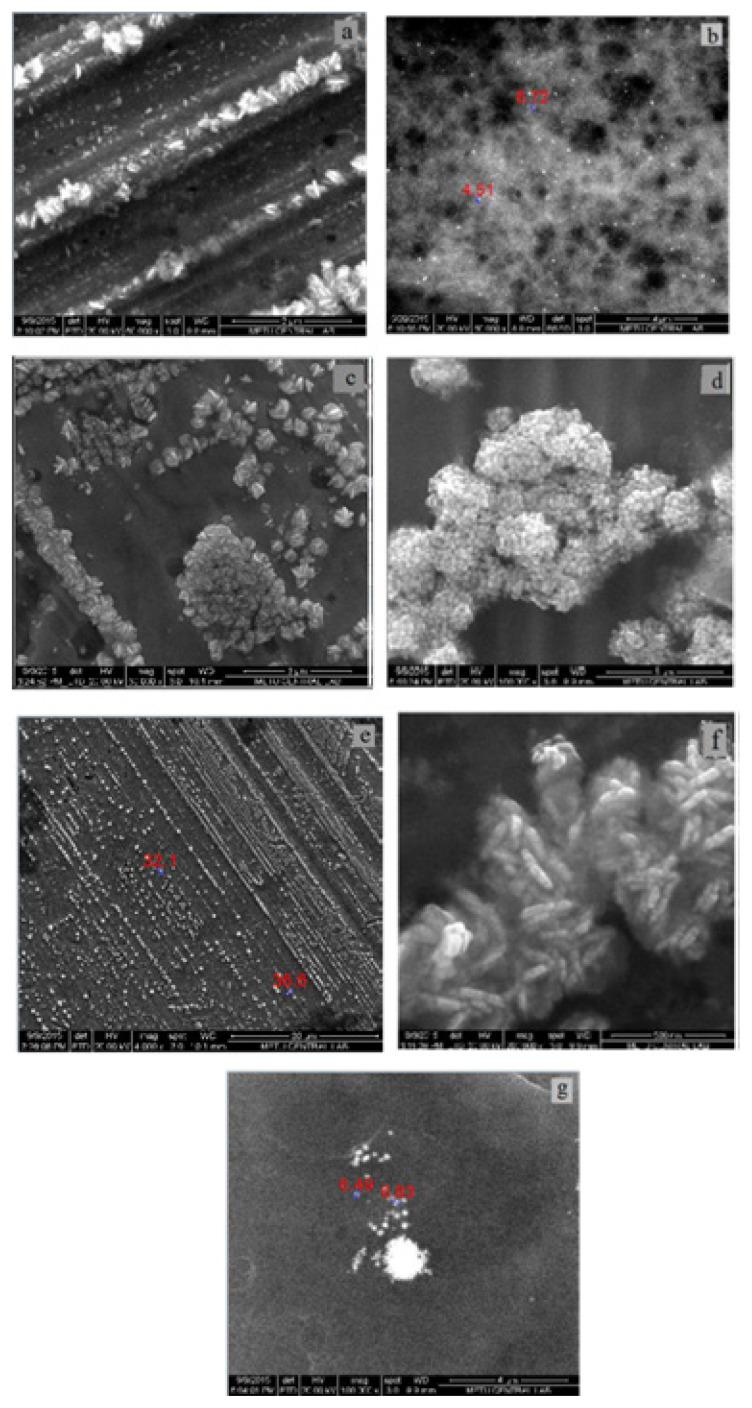
SEM images of AgNp synthesized with a) LE, b) CS, c) MD, d) CP, e) AVR, f) PC, and g) OVB bioextracts.

SEM images of AgNp synthesized using seven different bioextracts are given in Figure 12. When SEM images of AgNp were examined, it was seen that Nps have different shapes and structures. AgNps in different shapes and sizes could be synthesized according to the type of bioextract used and the experimental conditions. It has been observed that the largest spherical AgNps synthesized by AVR bioextract are 30–35 nm. However, the sizes of AgNps synthesized by CS and OVB bioextracts, in which deviations from sphericity were observed, are 6–10 nm. For spherical AgNp synthesized with CP bioextract, aggregation was observed intensely, so measurements could not be taken. AgNp synthesized with LE, MD and PC bioextracts showed rod structure.

#### 3.2.8. EDX analysis of AgNp

EDX analysis for the characterisation of AgNp synthesized with seven selected bioextracts was carried out separately. An example of EDX results was given in Figure 8b for AgNp synthesized with CP. Due to the SPR of AgNp, characteristic peaks were generally observed at approximately 3 keV (Figure 8b) [44].

When EDX spectrum (Figure 8b) was examined, strong peaks around 3.00 keV showed that AgNp was successfully synthesized [44]. Along with the peaks indicating the formation of AgNp, weak characteristic peaks of C from bioextracts were also observed. The peak of Cu is due to the copper bands used to provide conductivity while preparing the sample. It was observed that the results obtained from EDX spectra of AgNp synthesized with other bioextracts were similar.

#### 3.2.9. XPS analysis of AgNp

XPS spectrum of AgNPs synthesized using LE extract is given in Figure 9b as an example. For AgNp, the characteristic peaks at 367 and 374 eV binding energies of the electrons in the 3d_5/2_ and 3d_3/2_ spins of Ag^0^ showed that AgNps were successfully synthesized [45]. The binding energy at 367 eV is for Ag 3d_5/2_ component in an organic structure to which AgNps are bound (Figure 9b). The binding energy of 374 eV for Ag 3d_3/2_ component is an indication of the presence of metallic silver atoms (Figure 9b). These characteristic peaks were also obtained in XPS spectra of AgNp synthesized with other bioextracts.

#### 3.2.10. FTIR spectra of AgNp

**Figure 13:**
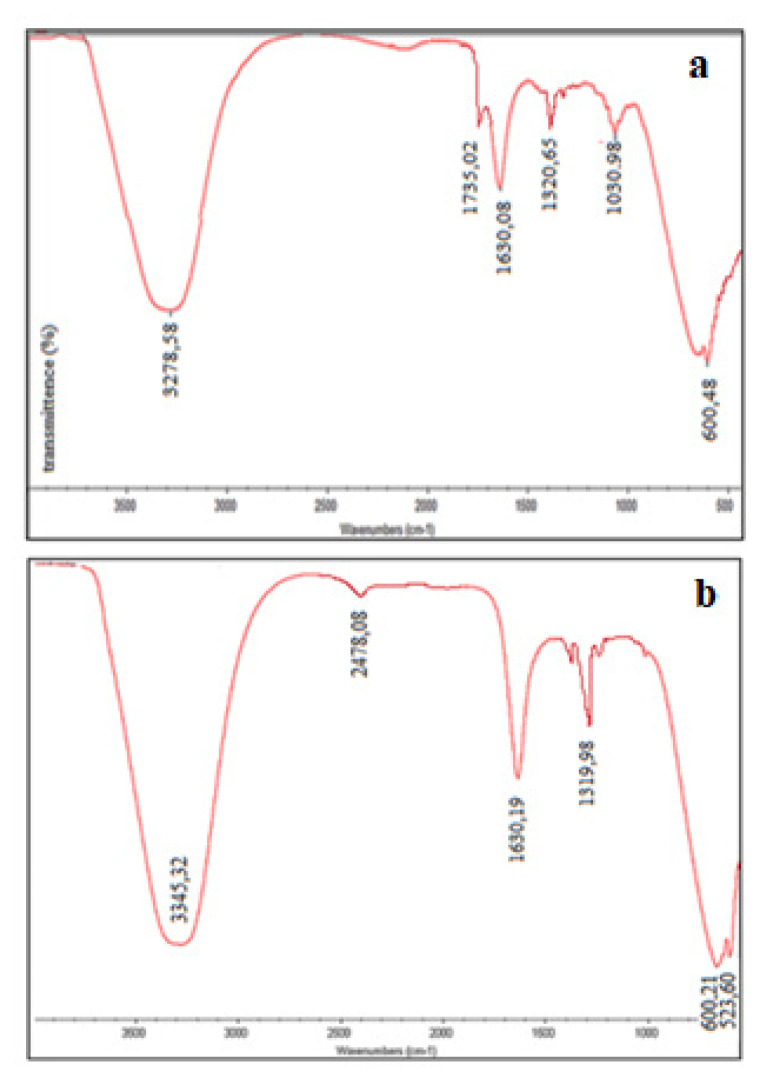
FTIR spectra of MD bioextract (a) and AgNp solution (b).

Typical FTIR spectra for the characterisation of synthesized AgNp are given in Figure 13. In this case, FTIR spectrum of the solution containing AgNp was compared with that of MD bioextract.

The vibration bands observed at 3345.32, 2478.08, and 1630.19 cm^−1^ in the FTIR spectrum of solutions containing AgNp synthesized by MD bioextract are the stretching of OH, CH and –C=C– covalent bonds, respectively (Figure 13b). The band observed at 1320.65 cm^−1^ in the FTIR spectrum of the bioextract (Figure 13a), due to the vibrational movement of >C=O and CO groups, shifted to 1319.98 cm^−1^ with the formation of AgNp and the >C=O band at 1735.02 cm^−1^ was not observed (Figure 13b). These changes can be interpreted that AgNps bind to –COO groups. However, the existence of the band assigned to the vibrational movement of Ag–O ionic bond groups was observed at 523.60 cm^−1^ [40].

Especially at FTIR spectra of Au and Ag, it is thought to compare these two formations by giving the spectra of Nps synthesized from the same extract. While shifting of >C=O and amide bands is important at FTIR characterisation of AuNps, only the shift of the band which is belong to the >C=O group in the case of AgNps, is important. From these observations, it is clear that the agents (responsible for reduction and stabilization) involved in the synthesis of Au and Ag-Nps are amines, ketones, aldehydes or carboxylic acids, which are the metabolite wastes of the bioextract.

### 3.3. Characterisation of MNp modified graphite electrodes (AuNp/GE and AgNp/GE)

By using AuNp and AgNp synthesized from seven different bioextracts, the surfaces of PGEs were modified. With this study, it has been demonstrated that AuNp and AgNp synthesized by a simple, inexpensive and environmentally friendly green synthesis method can be easily used in electrode modification. For this purpose, PGEs were modified with AuNp and AgNp which synthesized by LE bioextract at room temperature. The electrochemical characterisation of the prepared AuNp/GE and AgNp/GE was performed using CV and EIS techniques.

#### 3.3.1. Electrochemical characterisation of AuNp/GEs

**Figure 14:**
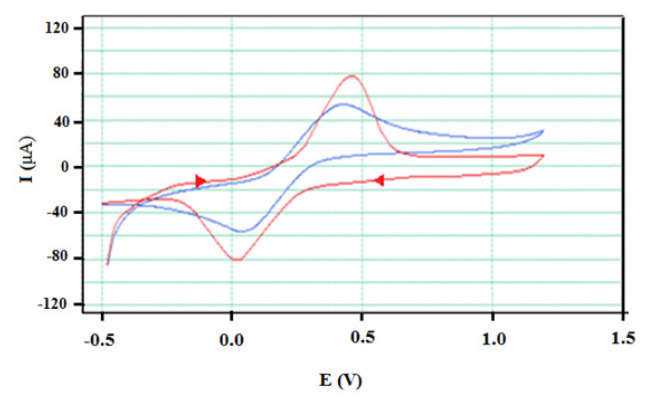
Cyclic voltammograms of [Fe(CN)_6_]^4^^−^ and [Fe(CN)_6_]^3^^−^ redox couple on bare GE (blue line) and AuNp/GE (red line) in 0.1 M KCl supporting electrolyte (Ag/AgCl reference electrode, Pt wire counter electrode and scan rate of 100 mVs^−^^1^).

**Figure 15:**
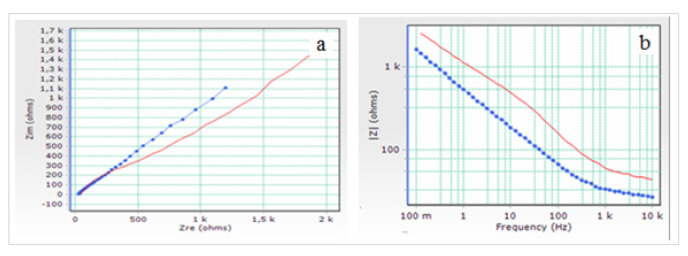
a) Nyquist and b) Bode impedance plots of [Fe(CN)_6_]^4^^−^ and [Fe(CN)_6_]^3^^−^ redox couple on bare GE (red line) and AuNp/GE (blue line) in 0.1 M KCl supporting electrolyte.

Cyclic voltammograms and EIS spectra of 1 × 10^−3^ M [Fe(CN)_6_]^4−^ and [Fe(CN)_6_]^3−^ redox pair on bare GE and the prepared AuNp/GE in 0.1 M KCl supporting electrolyte were given in Figures 14 and 15.

As seen in Figure 14, when the oxidation and reduction peak potentials and peak currents at cyclic voltammograms of [Fe(CN)_6_]^4−^ and [Fe(CN)_6_]^3−^ redox couple on bare GE and modified AuNp/GE are compared, it was observed that the most significant change on AuNp/GE was the increase in peak current. This finding shows that AuNp/GE is more sensitive for the redox couple than bare GE. As the scan rate (*v*) increases (50–250 mV s^−1^), the peak current increases (log *I_pc_* = 0.51 log *v +* 0.92), indicating that reduction and oxidation on the electrode surface are diffusion-controlled [46, 47].

Another study for the electrochemical characterisation of AuNp/GE was carried out by the EIS method. By interpreting the values of the electrochemical parameters obtained in this method, detailed information about the characterisation of this modified electrode was obtained. In Figure 15, EIS spectrum of [Fe(CN)_6_]^4−^ and [Fe(CN)_6_]^3−^ redox couple on AuNp/GE in 0.1 M KCl supporting electrolyte was compared with that obtained on bare GE.

As seen in Figure 15a, the Nyquist curve consists of two regions. At the beginning there is a slight semicircular region, followed by a linear part. The semicircular region describes the charge transfer resistance and diffusion layer resistance. The presence of the linear region indicates that the event on the surface is diffusion-controlled [48]. This finding was also supported by CV studies. The electrochemical parameters of the equivalent circuit from the a and b curves given in Figure 15 were determined with ZsimpWin software of Princeton Applied Research and the values of the calculated electrochemical parameters are given in Table 2.

From Nyquist curves, electrochemical parameters such as Warburg impedance, electron transfer rate constant (*k_ct_*), electron transfer resistance (*R_ct_*), solution resistance (*R_s_*), capacitance (*C_dl_* and *Q_dl_*) and constant phase element (*CPE*) can be obtained.

As seen in Figure 15 and Table 2, electron transfer resistance (*R_ct_*) values were found to be 1.03×10^4^ and 1.25×10^3^ Ohm/cm^2^ for bare GE and AuNp/GEs, respectively. AuNp/GE has an excellent electrocatalytic activity with an increase in the electron transfer rate on the electrode surface, because of the fact that it has a much lower *R_ct_* value than bare GE electrode [46,49,50]. When the *R_s_* values, expressing the solution resistance are examined, it was seen that GE > AuNp/GE. This indicates that the microscopic surface of AuNp/GE has increased [46,51,52].

The double layer capacitance *Q_dl_* is a constant phase element and represents a frequency dependent electrochemical phenomenon. When the electrode surface is rough and porous, the electronic properties of the interface cannot be described well enough with a capacitance element and a constant phase element *Q_dl_* has to be substituted for *C_dl_* [46, 52–54].

The impedance of CPE is determined by the following equation:


(1) $${\text{Z}}_{\text{CPE}}(\omega )={\text{Z}}_{0}{\left(\text{j}\omega \right)}^{-\alpha },$$

where α is phase in the range 0 < α < 1, *j* is the imaginary number, Z_0_ is a constant and ω is the angular frequency [55]. When α value is close to 1, CPE behaves like an ideal capacitor and becomes *Q_dl_* ≡ *C_dl_* [53,56].

In this study, *Q_dl_* was used as the capacitance. CPE values for bare GE and AuNp/GE from the ZSimpWin simulation program were found to be 7.13 × 10^−5^ and 1.02 × 10^−4^ F/cm^2^ respectively (Table 2). The reason for the increase in this value can be explained by the increase in dielectric constant, conductivity and porosity [57]. When α is 0.5 in the above equation, it behaves like Warburg impedance [58]. The reason for the formation of Warburg (*W*) impedance is diffusion. The Warburg impedance depends on the frequency at the potential deviations. The Warburg impedance is small because the diffusion reactants do not move very fast in the high frequency region. From Table 2, it was seen that AuNp/GE > GE for *W* value. The fact that this value was higher at the Np modified electrode showed that the movements of the reactants accelerated, thus increasing the diffusion. The *k_ct_* was calculated using the following equation:


(2) $$k_{ct}=\text{RT}/{\text{n}}^{2}{\text{F}}^{2}R_{ct}AC_{redox},$$

where *A* is the geometric area of the electrode surface and *C_redox_* is the concentration of the redox couple [59]. A noticeable increase was observed in the *k_ct_* of AuNp/GE compared to bare GE (Table 2). This shows that the electron transfer process on AuNp/GE is easier and faster.

#### 3.3.2. Electrochemical characterisation of AgNp/GEs

**Figure 16:**
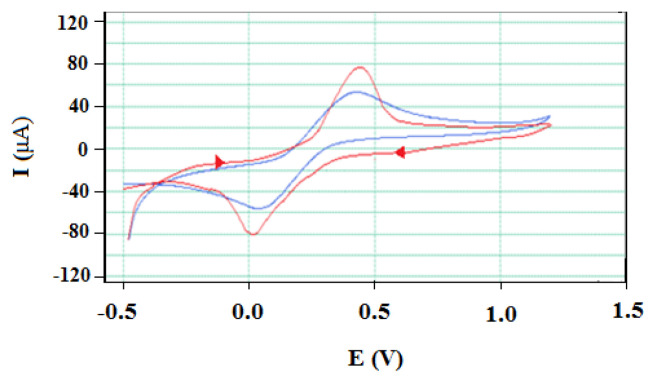
Cyclic voltammograms of 1 × 10^−^^3^ M [Fe(CN)_6_]^4^^−^ / [Fe(CN)_6_]^3^^−^ redox couple on bare GE (blue line) and AgNp/GE (red line) in 0.1 M KCl supporting electrolyte.

**Figure 17:**
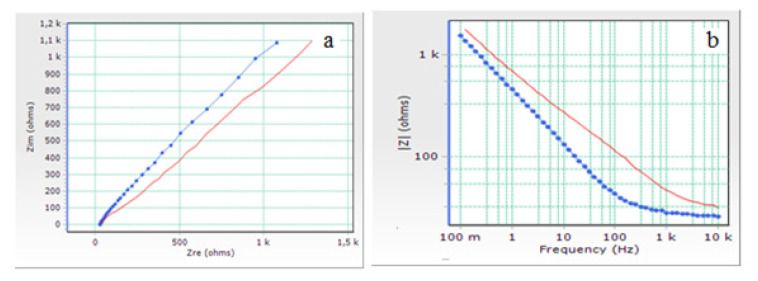
a) Nyquist and b) Bode impedance plots of [Fe(CN)_6_]^4^^−^ / [Fe(CN)_6_]^3^^−^ redox couple on bare GE (red line) and AgNp/GE (blue line) in 0.1 M KCl supporting electrolyte.

The cyclic voltammograms and EIS spectra of 1 × 10^−3^ M [Fe(CN)_6_]^4−^ / [Fe(CN)_6_]^3−^ redox pairs on bare GE and the prepared AgNp/GE in 0.1 M KCl supporting electrolyte are exhibited in Figures 16 and 17, respectively.

It was observed that there was a significant increase on AgNp/GE compared to bare GE in the oxidation and reduction peak currents of 1 × 10^−3^ M [Fe(CN)_6_]^4−^ / [Fe(CN)_6_]^3−^ redox couple in 0.1 M KCl supporting electrolyte (Figure 16). This finding was supported by further investigation of the characterisation of AgNp/GEs using EIS technique. The curves and calculated electrochemical parameters of EIS studies carried out for this purpose are given in Figure 17 and Table 2, respectively.

When Figure 17 and Table 2 are examined, the decrease in *R_s_* and *R_ct_* values and however the increase in *k_ct_*, CPE and *W* values clearly show that the surface of GE has been successfully modified with AgNp and its electrochemical properties have been greatly improved. Thus, it has been shown that AgNp/GEs have better conductivity and higher electron transfer rate, excellent electrocatalytic activity, a more porous structure, faster and easier diffusion.

## 3. Conclusion

In this study, green synthesis of AuNp and AgNp was carried out using LE, CS, MD, CP, AVR, PC and OVB bioextracts. As a result of this study, green synthesis of both Au and Ag-Nps was carried out with the seven bioextracts, the total fourteen MNps were obtained. From the obtained experimental data, it has been determined that AuNp and AgNp can be easily synthesized. In the second stage of the study, modified AuNp/GE and AgNp/GE were prepared using the synthesized AuNp and AgNp. CV and EIS techniques were used for the electrochemical characterisation of these modified electrodes. The obtained electrochemical parameters showed that *R_s_* and *R_ct_* decreased, whereas *k_ct_, CPE* and *W* values increased for modified AuNp/GE and AgNp/GE with compared to bare GE. These findings showed that AuNp/GE and AgNp/GE with better conductivity, higher electron transfer rate and better electrocatalytic activity were prepared as a result of successful electrode modification. Electron transfer property of the GE was improved by modification with green synthesized AuNp and AgNp, the modified electrodes to be good potential for electrochemical applications as electrochemical sensors.

The present study showed that green synthesized nanoparticles modified electrodes exhibit potential in bio/sensor preparation and electronic application. These experimental results will shed light on biosensor studies, applications in the field of biomedicine, drug development and nanomaterial development studies. It should not be forgotten that the uses of the bioextracts in the green synthesis of MNps are very important step for electroanalytical applications.

## Figures and Tables

**Table 1:** Optimum experimental conditions for the green synthesis of AuNp and AgNp.

MNp	Bioextract	Colour change		pH	Ratio of bioextract to metal	Reaction time (min)
		Before	After			
*AuNp*	LE	Light orange	Intense dark pink	10.0	3:7	Immediately
	CS	Colourless	Intense dark pink	8.5	5:5	Immediately
	MD	Yellow	Brownish violet	10.0	3:7	Immediately
	CP	White	Pink	9.0	3:7	Immediately
	AVR	Yellow	Red	10.5	5:5	Immediately
	PC	Yellow	Brown	11.0	3:7	Immediately
	OVB	White	Dark pink	8.0	5:5	Immediately
*AgNp*	LE	Light orange	Brown	9.0	5:5	1–5
	CS	Colourless	Brown	9.5	3:7	Immediately
	MD	Yellow	Brown	9.5	3:7	Immediately
	CP	White	Intense dark brown	10.0	5:5	5–15
	AVR	Yellow	Dark brown	10.5	3:7	Immediately
	PC	Yellow	Brown	9.0	5:5	3–10
	OVB	White	Intense dark brown	9.5	3:7	Immediately

**Table 2:** Electrochemical parameters of EIS spectra of [Fe(CN)_6_]^4^^−^ and [Fe(CN)_6_]^3^^−^ redox couple on GE, AuNp/GE and AgNp/GE in 0.1 M KCl supporting electrolyte.

	*R(C(RW))*					

	*R* *_s_* * (Ωcm* *^−2^* *)*	*CPE/Y_0_ (S/s^a^) a* ≈1 (Fcm^−2^)	*R* *_ct_* * (Ωcm* *^−2^* *)*	*W /Y* *_0_* * (S/s* *^0.5^* *) (Ωcm* *^−2^* *)*	*χ* *^2^*	*k* *_ct_* * (cm s* *^−1^* *)*

GE	6.46 × 10^2^	7.13 × 10^−5^	1.03 × 10^4^	5.27 × 10^−3^	1.29 × 10^−2^	4.28 × 10^−3^
AuNp/GE	3.38 × 10^2^	1.02 × 10^−4^	1.25 × 10^3^	7.35 × 10^−3^	1.50 × 10^−3^	3.53 × 10^−2^

GE	4.44 × 10^2^	4.73 × 10^−5^	8.16 × 10^3^	7.98 × 10^−3^	3.65 × 10^−3^	5.40 × 10^−3^
AgNp/GE	3.52 × 10^2^	8.64 × 10^−5^	1.29 × 10^3^	1.06 × 10^−2^	1.25 × 10^−3^	3.43 × 10^−2^

* EIS parameters have been normalised according to the geometric electrode area.
